# Identification of YfiH and the Catalase CatA As Polyphenol Oxidases of *Aeromonas media* and CatA as a Regulator of Pigmentation by Its Peroxyl Radical Scavenging Capacity

**DOI:** 10.3389/fmicb.2017.01939

**Published:** 2017-10-05

**Authors:** Baozhong Chai, Yunqian Qiao, He Wang, Xiaoming Zhang, Jiao Wang, Choushi Wang, Ping Zhou, Xiangdong Chen

**Affiliations:** ^1^State Key Laboratory of Virology, College of Life Sciences, Wuhan University, Wuhan, China; ^2^Analytical and Testing Center, College of Chemistry and Molecular Sciences, Wuhan University, Wuhan, China; ^3^China Center for Type Culture Collection, Wuhan, China

**Keywords:** melanin, pigmentation, bioinformatic analysis, polyphenol oxidase, catalase

## Abstract

Pyomelanin is the major constituent of pigment in melanogenic *Aeromonas* strains of bacteria. However, eumelanin, synthesized from tyrosine via L-DOPA and polyphenol oxidases (PPOs), may also be present in this genus since L-DOPA is frequently detected in culture fluids of several species. To address this question, we used a deletion mutant of *Aeromonas media* strain WS, in which pyomelanin synthesis is completely blocked under normal culture conditions. When tyrosine was supplied to the medium, we observed residual melanin accumulation, which we interpret as evidence for existence of the DOPA-melanin pathway. We traced enzymatic activity in this bacterium using native-polyacrylamide gel electrophoresis. Two PPOs: YfiH, a laccase-like protein, and CatA, a catalase, were identified. However, neither protein was critical for the residual pigmentation in pyomelanin-deficient mutant. We speculate that eumelanin synthesis may require other unknown enzymes. Deletion of *yfiH* did not affect pigmentation in *A. media* strain WS, while deletion of the CatA-encoding gene *katE* resulted in a reduction of melanin accumulation, but it started 9 h earlier than in the wild-type. Since catalases regulate reactive oxygen species levels during melanogenesis, we speculated that CatA affects pigmentation through its peroxyl radical scavenging capacity. Consistent with this, expression of the catalases Hpi or Hpii from *Escherichia coli* in the *katE* deletion strain of *A. media* strain WS restored pigmentation to the wild-type level. Hpi and Hpii also exhibited PPO activity, suggesting that catalase may represent a new class of PPOs.

## Introduction

Melanin refers to a group of a polyphenolic pigments derived from the oxidation and polymerization of tyrosine in animals or from phenolic compounds in lower organisms (d’Ischia et al., 2013). Melanin is regarded as one of the most enigmatic group of pigments in nature. These pigments are usually classified into several categories based on the intermediates of melanogenesis: melanin derived from L-3,4-dihydroxyphenylalanine (DOPA) is referred to as DOPA-melanin, melanin derived from homogentisic acid (HGA) is HGA-melanin, neuromelanin is derived from dopamine or other catecholamine precursors, and melanin derived from 1, 8-dihydroxynaphthalene is referred to as DHN melanin. All of these pigments are believed to be associated with survival mechanisms, for example, offering protection from ultraviolet (UV) light in *Bacillus thuringiensis* or involved in the symbiotic relationship of soil bacteria such as *Azospirillum* and *Rhizobium* sp. with plants (Piñero et al., 2007; Herter et al., 2011; Mohamed et al., 2012). The widespread existence and pleiotropic functions of melanin in nature suggest an evolutionary importance of melanogenesis (Plonka and Grabacka, 2006).

Bacteria usually produce DOPA-melanin and HGA-melanin. DOPA-melanin has been identified in a wide range of bacterial genera, including *Streptomyces*, *Bacillus*, *Marinomonas*, *Ralstonia*, *Rhizobium*, *Verrucomicrobium*, *Thermomicrobium*, and *Sinorhizobium* (Fairhead and Thöny-Meyer, 2012). The polyphenol oxidases (PPOs) responsible for DOPA-melanin production are usually of the multi-copper oxidase type, usually tyrosinase (EC 1.14.18.1) but in some cases laccases are responsible (EC 1.10.3.2; Fairhead and Thöny-Meyer, 2012). Some bacteria, notably *Vibrio cholera, Shewanella colwelliana*, *Pseudomonas putida*, and *Pseudomonas aeruginosa*, synthesize pyomelanin via what is known as the HGA pathway (Kotob et al., 1995; Arias-Barrau et al., 2004). In the HGA pathway, 4-hydroxyphenylpyruvate is converted to HGA by 4-hydroxyphenylpyruvate dioxygenase (HppD), followed by auto-oxidation and polymerization of HGA to melanin (Claus and Decker, 2006).

Production of melanin has been described in some species of the bacterial genus *Aeromonas*, including *Aeromonas salmonicida* and *A. punctata* (Abbott et al., 2003). Melanin biosynthesis is, in fact, a key differential characteristic in the taxonomy of this genus. It is an important pathogen of fish and other cold-blooded species, and an etiologic agent of a variety of infectious complications in humans (Janda and Abbott, 2010).

We recently showed that pigmentation of *A. media* strain WS, a high-melanin-yielding strain isolated from East Lake, Wuhan, China (Wan et al., 2007), is largely due to the production of pyomelanin through HGA. Deletion of *hppD*, which encodes the key enzyme 4-hydroxyphenylpyruvate dioxygenase, completely blocked pigmentation of the cultured bacterium. It has also been observed that the HGA biosynthesis pathway is widely distributed in *Aeromonas*, and has been observed in both pigmented and non-pigmented species (Wang et al., 2015). It is important to note, though, that the melanin is also synthesized by the DOPA-based pathway in many *Aeromonas* species, if not all of them. This conclusion is based on the detection of the characteristic intermediate L-DOPA in several melanogenic *Aeromonas* strains (Aurstad and Dahle, 1972; Donlon et al., 1983; Gibson and George, 1998; Wan et al., 2007). The conclusion is further supported as proteins unrelated to the HGA pathway regulate melanogenesis in *Aeromonas*, albeit by unknown mechanisms. An example of such a protein is the PilF protein in *A. veronii* strain B565, which is an outer membrane protein required for pilus biogenesis (Abolghait, 2013).

It appears, then, that the metabolism and synthesis of melanin in *Aeromonas* are complex. Because no typical bacterial tyrosinase is present in the documented *Aeromonas* genome sequences (Chai et al., 2012), we set out to identify the PPOs that may be responsible for DOPA-melanin production in *A. media* strain WS. Two PPOs were identified by tracing enzymatic activities with native-polyacrylamide gel electrophoresis (PAGE). However, neither of them was critical for the formation of DOPA-melanin, suggesting that DOPA-melanin production in this strain does not require PPO. One of the enzymes, a catalase, affected the total melanogenesis by its peroxyl radical scavenging capacity. We hypothesized that oxidative stress regulates melanogenesis.

## Materials and Methods

### Bacterial Strains, Plasmids and Culture Conditions

The bacterial strains and plasmids used in this study are listed in **Table 1**. The wild-type and mutant strains of *A. media* strain WS were grown at 30°C in Luria–Bertani (LB) medium supplemented with chloramphenicol (30 μg/mL) or ampicillin (100 μg/mL) when necessary. *Escherichia coli* were routinely grown at 37°C in LB medium and supplemented with kanamycin (50 μg/mL) or chloramphenicol (30 μg/mL) when they were harboring plasmids. Bacterial strain *E. coli* S17-1 (λ*pir*) and plasmid pDM4 were donated by Prof. Shiyun Chen of Wuhan Institute of Virology, Chinese Academy of Sciences, Wuhan, China. Plasmid pBBR1MCS-5 was kindly donated by Prof. Zhixiong Xie of Wuhan University, Wuhan, China.

**Table 1:** Bacterial strains and plasmids used in this study.

Strains or plasmids	Genotype and/or characteristic(s)	Reference
**Strain**		
WS (Wild-type)	High-melanin-yielding isolate, *Amp^r^*	Wan et al., 2007
WS Δ*hppd*	WS with a deletion of *hppd*	Wang et al., 2015
WS Δ*yfiH*	WS with a deletion of *yfiH*	This study
WS Δ*katE*	WS with a deletion of *katE*	This study
WS Δ*yfiH*Δ*katE*	WS Δ*yfiH* with a deletion of *katE*	This study
DH5α	*supE*44 Δ*lacU*169(φ80*lacZ*Δ*M*15) *hsdR*17 *recA*1 *endA*1 *gyrA*96 *thi*-1 *relA*1	Our lab
BL21 (DE3)	*F^-^ ompT hsdS(rBB-mB-) gal dcm*(DE3)	Our lab
S17-1 (λ*pir*)	*thi pro hsdR hsdM*1 *recA* RP4-2-Tc::Mu-Km::Tn*7*	Simon et al., 1983
**Plasmid**		
pUC18	Cloning vector, *Amp^r^*	Our lab
pDM4	R6Kγ *ori* (requires π), *oriT* of RP4; *Cm^r^*	Simon et al., 1983
pDMM1	0.5-kb fusion PCR fragment containing Δ*yfiH* cloned into pDM4; used to make the WS Δ*yfiH* mutant	This study
pDMM2	0.9-kb fusion PCR fragment containing Δ*katE* cloned into pDM4; used to make the WS Δ*katE* mutant	This study
pBBR1MCS-5	Mobilizable broad-host-range vector, *Gm^r^*	Kovach et al., 1995
pBBR1MCS-5-*katE*	Vector for *in vivo* complementation of *katE* mutant	This study
pBBR1MCS-5-*hpi*	Vector for *in vivo* complementation of *katE* mutant using *hpi* gene from *E. coli*	This study
pBBR1MCS-5-*hpii*	Vector for *in vivo* complementation of *katE* mutant using *hpii* gene from *E. coli*	This study
pET-26b (+)	Expression vector, *Kan^r^*	Our lab
pET-CatA	*katE* cloned into the *Nde*I/*Xho*I site of pET26-b(+)	This study
pET-Hpi	*hpi* cloned into the *Nde*I/*Xho*I site of pET26-b(+)	This study
pET-Hpii	*hpii* cloned into the *Nde*I/*Xho*I site of pET26-b(+)	This study


### Identification of the DOPA-Melanin Precursor by High-Performance Liquid Chromatography (HPLC) Analysis

Bacterial culture was taken 24 h after the start of the cultures and centrifuged at 13,000 × *g* for 10 min to remove bacterial cells. The supernatant was immediately acidified with 0.1 volume of glacial acetic acid. After centrifugation, the sample was diluted threefold with 10 mM acetic acid and filtered through a 0.45-μm filter (Millipore, MA, United States). Chromatographic separation was achieved on an Agilent Eclipse column, which was fitted with C18 reverse phase column (250 mm × 4.6 mm i.d., 5 μm). Peaks eluting from the column were detected with a photodiode array detector (G1315B) at 260 nm, the absorption maxima of L-DOPA. L-DOPA (Sigma, United States) was used as the standard.

### Purification and Characterization of Melanin from *A. media* Strain WS

The melanin was prepared from *A. media* strain WS. Synthetic DOPA-melanin (Sigma, United States) was used as the standard. Bacteria were cultivated in LB medium at 30°C with shaking at 200 rev min^-1^. After 72 h, the culture was centrifuged at room temperature for 15 min at 13,000 × *g* and the supernatant was filtered through a 0.45-μm filter (Millipore, MA, United States). The pH of the cell-free filtrate was adjusted to 3.0 with HCl and shaken at 25°C to hydrolyze the proteins bound with melanin. The filtrate was centrifuged at 13,000 × *g* and the pellet, containing the melanin, was dissolved in NaOH solution (pH 9.0). The precipitation process was repeated for three times. The melanin deposition was washed with double-distilled water and dried under a vacuum.

The melanin samples for Fourier transform infrared spectroscopy (FT-IR) analysis were prepared by mixing the melanin with potassium bromide powder. Analysis was carried out on a Nicolet 5700 FT-IR Spectrometer (Madison, WI, United States). The spectra recorded over a range of 4,000–400 cm^-1^ with a resolution of 4 cm^-1^.

The melanin samples (5 mg) were dissolved in 4 mL of methyl alcohol:acetonitrile (v/v 3:1). The electrospray ionization mass spectrometry (ESI-MS) of melanin samples were performed on LCQ Advantage instrument with an ESI source (4.8 KeV). The m/z range scanned in the MS measurements was from 50 to 1,000 or 2,000.

### Polyphenol Oxidase Activity Assay by Native-PAGE

The PPO activity assay in this study was performed on native-PAGE. Briefly, bacterial strains were cultivated for 18 h at 30°C. The cells were harvested by centrifugation at 13,000 × *g* for 10 min, washed and resuspended in 50 mM Tris-HCl buffer (pH 7.0) and lysed by sonication. Cell lysates were centrifuged at 13,000 × *g* for 20 min at 4°C to remove cell debris. Electrophoresis was performed at 4°C on polyacrylamide gels, without sodium dodecyl sulfate (SDS) and β-mercaptoethanol to retain the activity of enzymes, at a constant current of 20 mA for about 8 h. PPO activity was assessed after incubating the gel at 30°C in 50 mM Tris-HCl buffer (pH 8.0) plus 5 mM L-DOPA for 30 min with gentle shaking.

### Polyphenol Oxidase (PPO) Analysis on the Genome Sequence

Based on the draft genome sequence of *A. media* strain WS, the whole genome was sequenced using a combination approach of Roche 454 GS-FLX Titanium platform and PacBio RS II system. *De novo* genome assembly of PacBio reads was performed by Velvet 1.2.03 and Illumina reads sequences were used to estimate gap sizes of adjacent contig pairs and to evaluate correct assembly throughout the project. Open reading frames (ORFs) were predicted with Glimmer and annotated using KEGG, UniProt, and COG (Clusters of Orthologous Group) databases.

The conserved domains of tyrosinase or laccase, which are essential to DOPA melanin formation in bacteria, were screened online at http://www.ncbi.nlm.nih.gov/Structure/cdd/cdd.shtml. The potential proteins were predicted by searching the whole genome against these conserved domains.

### Identification of Polyphenol Oxidase Using Liquid Chromatography-Tandem Mass Spectrometry Technology

Two PPO activity bands were observed by native-PAGE assay. After the identification of one PPO (PPO1) in *A. media* strain WS via bioinformatic method, the PPO activity of the other PPO (PPO2) was performed on native-PAGE after precipitation with ammonium sulfate at 35% saturation. The staining band of PPO2 was then cut from native-PAGE gel and characterized by liquid chromatography using matrix-assisted laser desorption/ionization/time-of-flight (MALDI-TOF/TOF) mass spectrometer at HuaDa (Guangdong, China).

Peak lists from LC-MS/MS experiments were generated by the LCQ software Bioworks Browser via TurboSEQUEST. The MASCOT search engine^1^ was used for the protein database search using the completed genomic sequence of *A. media* strain WS. The output of the MASCOT search results was extracted to an Excel file to provide a list of proteins identified by the peptides. Finally, candidate proteins exhibiting PPO2 activity were manually examined by reaction analysis of each protein using KEGG database.

### Generation of Deletion Mutants and Complementation of CatA

The deletion mutants of PPO activity proteins, designated as WS Δ*yfiH*, WS Δ*katE*, and WS Δ*yfiH*Δ*katE*, were made by homologous recombination. For all constructions, a suicide vector pDM4 containing fusion PCR fragments located upstream and downstream of *yfiH* or *katE* (primer pairs for PCR; see Supplementary Table S1) with in-frame deletions were used. To introduce these plasmids into *A. media*, conjugal matings were done as follows: the knock-out vectors pDMM1, pDMM2, and pDMM3 were introduced into *E. coli* S17-1λ *(pir)* and the resultant strains mated with *A. media*. Transconjugants were selected by utilizing the chloramphenicol resistance gene located on the suicide plasmid and confirmed by PCR. A single transconjugant of each strain was grown on 15% sucrose plate without antibiotics, allowing the occurrence of the second cross that replaced the wild-type allele with the mutant one. The transconjugants were sensitive to sucrose due to the synthesis of levans (toxic compounds to Gram-negative bacteria) catalyzed by levansuerase, the product of *sacB* located on pDM4. Thus, only the clones that exchanged for the second time and excised the plasmid-borne sequence could survive on these plates. The resulting clones that could not grow on plates with chloramphenicol were selected and confirmed by PCR and DNA sequencing.

To complement the function of CatA in WS Δ*katE*, the intact *katE* gene was amplified from chromosomal DNA of *A. media* strain WS using the primer pairs listed in Supplementary Table S1. The PCR product was cloned into the shuttle vector pBBR1MCS-5 (Kovach et al., 1995) and the resulting plasmid pBBR1MCS-5-*katE* was introduced by conjugation into the mutant strain WS Δ*katE*. The shuttle vector pBBR1MCS-5 was employed as well, to construct the complementary strain of *hpii* and *hpi* in WS Δ*katE*.

### Catalase Activity Assay

Catalase (CAT) activity was measured using an assay kit (Jiancheng, Nanjing, China), according to the manufacturer’s instructions. This kit utilizes the peroxidatic reaction of CAT for determination of enzyme activity. The hydrogen peroxide decomposition catalyzed by CAT was performed at 37°C for 1 min and then ammonium molybdate was added to terminate the reaction immediately. The residual hydrogen peroxide was monitored at 405 nm, the absorption maxima of the pale yellow complex produced by ammonium molybdate and hydrogen peroxide. CAT activity (U/mL) was defined as the consumption of H_2_O_2_ (μmol/sec) in buffer catalyzed by CAT at 37°C.

### Purification of the Active His_6_-Tagged CatA, Hpi, and Hpii

To construct the expression vector, the ORFs of CatA (1,449 bp), Hpi (2,181 bp) and Hpii (2,262 bp) were amplified from the genomes of *A. media* WS and *E. coli* BL21 (DE3) using primer pairs listed in Supplementary Table S1, respectively. The PCR products were inserted into the appropriate restriction enzyme sites of pET-26b (+) to yield pET-CatA, pET-Hpi, and pET-Hpii. *E. coli* BL21 (DE3), transformed with these plasmids were grown in LB medium containing 50 μg/mL kanamycin at 28°C and induced with 1 mM isopropyl-β-D-thiogalactoside at the exponential phase of growth (OD_600_ = 0.6). Purification of His_6_-tagged enzymes was carried out with Ni-NTA His●bind resins (Novagen). Bound proteins were eluted with a linear gradient from 20 to 500 mM imidazole. Fractions containing His_6_-tagged enzymes were determined by SDS-PAGE. Fractions containing the expected proteins were collected and dialyzed against 50 mM Tris–HCl buffer (pH 7.5). The concentration of purified proteins was determined using the Bradford assay (Bradford, 1976). The enzymatic analysis for PPO activity and CAT activity of active His_6_-tagged CatA, Hpi, and Hpii were performed as described above.

### Measurement of Melanin Production in PPO Mutants

Production of melanin in *A. media* strain WS and its PPO mutants (WS Δ*yfiH*, WS Δ*katE*, and WS Δ*yfiH*Δ*katE*) was monitored, using the method of Gibson (Gibson and George, 1998). To estimate the final melanin production, bacterial cultures were filtered through a 0.45-μm filter (Millipore, MA, United States) after 72 h of cultivation and the absorbance at 400 nm of the supernatants was measured. Growth of each strain was assessed by its OD at 600 nm. The capacity for melanin formation of wild-type and mutant strains was calculated using the OD_400_/OD_600_ value (Chatfield and Cianciotto, 2007); melanin production at various periods was recorded within 48 h of cultivation.

### Cell Viability Assay

Cell viability of *A. media* wild-type strain WS and its catalase-deficient strain, WSΔ*katE*, was assessed with 2,3,5-triphenyltetrazolium chloride (TTC) staining (Towill and Mazur, 1975). After 8 h in culture, equal inocula of each strain were transferred to LB medium grown at 30°C. Cells were collected at various times by centrifugation and suspension in 50 mM Tris-HCl buffer (pH 8.0) containing 0.05% TTC (Sigma, United States). Solutions were shaken in the dark for 12 h at 30°C and centrifuged at 13,000 × *g*. The precipitate was treated with 85% dimethyl sulfoxide (DMSO) to extract the triphenylformazan (TPF), the red TTC reduction product. After centrifugation, the absorption maximum of TPF was monitored at 485 nm. Growth of each strain was evaluated at each time point by measuring OD at 600 nm. The cell viability was calculated using the OD_485_/OD_600_ value. All assays were repeated for three times.

### Intracellular Reactive Oxygen Species (ROS) Assay

The fluorimetric probe dichloro-dihydrofluorescein diacetate (DCFH-DA, Sigma–Aldrich) was used for detection of reactive oxygen species (ROS). An RF-5301PC spectrofluorometer (Shimadzu, Inc., Kyoto, Japan) equipped with 1-cm quartz cell was used for fluorometric measurement. Bacterial cells were collected before and after pigmentation. Each sample was loaded with DCFH-DA to a final concentration of 20 μM and incubated in dark at 30°C for 40 min before the fluorometric measurement. Qualitative oxidative stress assay was determined by microscopy image analysis and quantitative determination was measured by fluorescence intensity of dichlorofluorescein (DCF) at an excitation wavelength of 499 nm and an emission wavelength of 521 nm. All assays were repeated at least four times.

## Results

### *A. media* Strain WS Produces DOPA-Melanin

Our previous work shows that HppD in the HGA pathway is crucial in melanin synthesis of *A. media* strain WS, a finding consistent with the observation that the WS Δ*hppD* mutant is completely incapable of pigment formation (Wang et al., 2015). However, melanin formation in this mutant was partially restored when the culture medium was supplemented with 2 mg/mL tyrosine (Supplementary Figure S1). We hypothesized that *A. media* strain WS is capable of synthesizing melanin via the DOPA-based pathway, using tyrosine as a substrate.

We tested this hypothesis in several ways. First, we purified the melanin produced by a culture of *A. media* strain WS using the purification procedure for bacterial melanin, and analyzed it with FT-IR. The absorption spectra are shown in Supplementary Figure S2A. The main absorption peaks of the purified melanin are quite similar to those of synthetic DOPA-melanin, which led us to suggest that DOPA-melanin was present in the sample from *A. media* strain WS.

**FIGURE 1:**
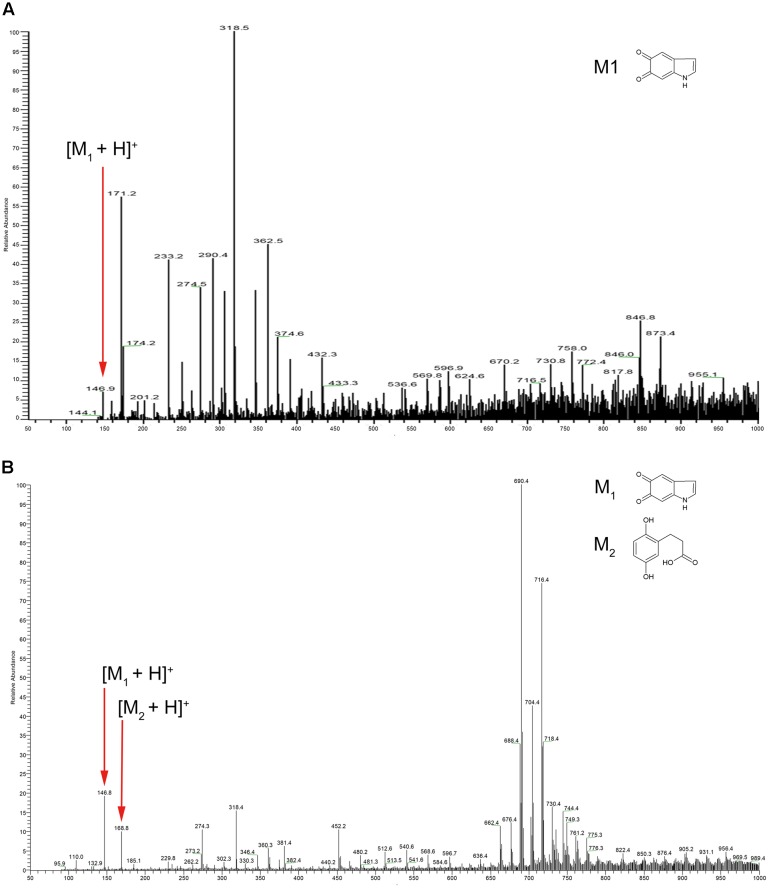
Electrospray ionization mass spectrometry (ESI-MS) profile of **(A)** synthetic DOPA-melanin and **(B)** melanin isolated from *Aeromonas media* strain WS. Note that the molecular ions at m/z 146, 274, and 318 were present in both; particularly, note m/z 146, which corresponds to indole-5, 6-quinone (marked with small red arrows).

We next employed mass spectrometry to compare the structure of the acid hydrolysis products of melanin from *A. media* strain WS (referred to here as WS melanin) and synthetic DOPA-melanin (obtained from L-DOPA). Both of these were analyzed by ESI-MS, and yielded similar spectra. Both samples displayed a protonated molecule [M+H]^+^ at *m/z* 147 (**Figure 1**), which corresponds to the characteristic monomer of DOPA-melanin, indole-5, 6-quinone (Costa et al., 1992). The ions at *m/z* 274, 318, and 717 in both MS spectra probably resulted from the fragmentation of a melanin polymer. These results provide further evidence supporting our hypothesis that DOPA-melanin was produced in *A. media* strain WS. Another observation from these mass spectrometry results is of interest. Pyomelanin, synthesized via the HGA pathway, is believed to be the main component of WS melanin (Wang et al., 2015). The protonated molecule [M+H]^+^ of HGA at *m/z* 169 was present in the spectra of WS melanin but not the spectra of synthetic DOPA-melanin, consistent with the presence of pyomelanin in the WS pigment sample also.

Our final test of the hypothesis was to perform HPLC analysis of pigment from *A. media* strain WS. The results are presented in Supplementary Figure S2B and show the presence of the precursor of DOPA-melanin, L-DOPA. This result is consistent with previous reports (Gibson and George, 1998; Wan et al., 2007). These results, together, lead to our conclusion that *A. media* strain WS is capable of synthesizing DOPA-melanin under certain culture conditions.

### Identification of Two Polyphenol Oxidases by Native-PAGE Assay

**FIGURE 2:**
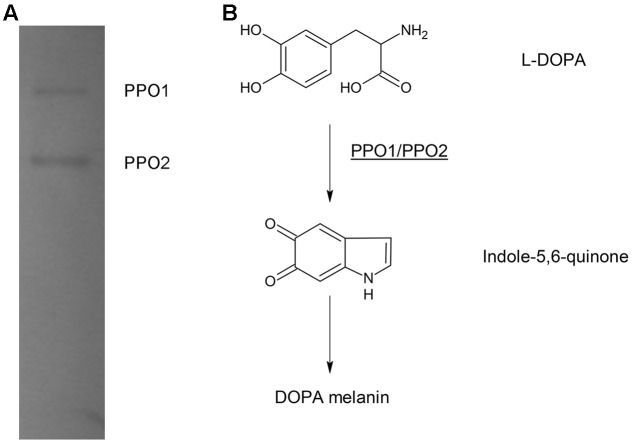
Electrophoretic analysis of polyphenol oxidase (PPO) activity in cell lysates of *A. media* strain WS. **(A)** The gel was stained with L-DOPA, the substrate of PPO. The two bands observed with the native-PAGE were designated as PPO 1 and PPO2. **(B)** Proposed DOPA-melanin pathway in *A. media* strain WS catalyzed by PPOs.

In bacteria, PPO, are usually responsible for DOPA-melanin synthesis. The PPOs are usually tyrosinases, but are occasionally laccase (Fairhead and Thöny-Meyer, 2012). We previously identified the PPO TyrA by native-PAGE at pH 9.0 from *A. media* strain WS (Wan et al., 2009). However, in this study, we noted that deletion of *tyrA* in the WS Δ*hppD* strain did not affect pigment production by the mutant when it was cultured in LB media supplemented with 2 mg/mL tyrosine. Further, the bands exhibiting PPO activity were still detected on native-PAGE (data not shown). From these observations, we conclude that TyrA is not a key PPO in *A. media* strain WS. Therefore, we turned our attention to identification of the PPOs that are responsible for the conversion of L-DOPA to DOPA-melanin in this strain. To this end, we performed a PPO activity assay in a gradient pH staining buffer. Cell lysate of *A. media* strain WS was subjected to native-PAGE staining with 5 mM L-DOPA as substrate. Two bands exhibiting PPO activity were detected at pH 7.5 or 8.0 (**Figure 2A**). We conclude that there are at least two PPOs that can convert L-DOPA to DOPA-melanin in WS, which we designated PPO1 and PPO2 here (**Figure 2B**).

### YfiH Is Responsible for PPO1 Activity in *A. media* Strain WS

The complete genome of *A. media* strain WS has been sequenced (GenBank sequence: CP007567.1), but no protein has been annotated as a PPO. It has been known for several decades that tyrosinases from bacteria, including *Streptomyces*, have the ability to convert L-DOPA into melanin (Fairhead and Thöny-Meyer, 2012), but no protein with a typical bacterial tyrosinase domain has been found in *A. media* strain WS by searching for the common central domain of tyrosinase (Pfam00264; Finn et al., 2006). Besides tyrosinase, proteins belonging to the multi-copper oxidase family are also involved in DOPA-melanin formation in bacteria, for example, laccase in *Sinorhizobium meliloti* (Claus and Decker, 2006). These proteins often contain multiple conserved domains of Cu-oxidase enzymes, notably, pfam00394, pfam07731, pfam07732, and pfam02578 (Messerschmidt and Huber, 1990; Messerschmidt et al., 1992; Page et al., 1999; Beloqui et al., 2006). One protein classified as K05810 in the KEGG Orthology (KO) database (protein SeqID: B224_5050) stood out when we screened all the predicted proteins against four conserved domains of multi-copper oxidase. Protein blast analysis of B224_5050 (gene name: *yfiH*) revealed that it belongs to the Cu-oxidase_4 family, which contains the pfam02578 conserved domain homologous to laccase.

**FIGURE 3:**
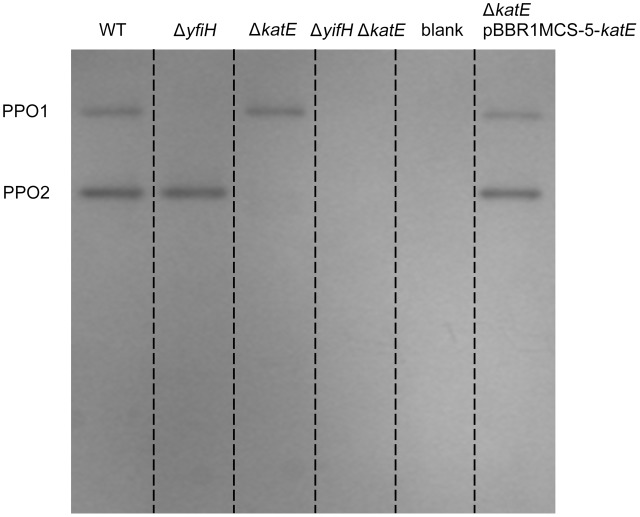
Polyphenol oxidase activity assay of PPO mutants and complementary strains on native-PAGE stained with L-DOPA. Gel lanes: (1) *A. media* strain WS (wild-type); (2) WS Δ*yfiH*; (3) WS Δ*katE*; (4) WS Δ*yfiH*Δ*katE*, (5) WS Δ*katE* complemented with an intact *katE* gene in trans on the plasmid pBBR1MCS-5.

To determine whether *yfiH* encodes a PPO, an in-frame deletion mutant of *yfiH* was constructed. Cells of both *A. media* strain WS (wild type) and mutant WS Δ*yfiH* were collected at the same period, and the cell lysates were subjected to native-PAGE staining with 5 mM L-DOPA to detect the diphenol oxidase activity. As shown in **Figure 3**, the band corresponding to PPO1 disappeared in WS Δ*yfiH*, indicating that the laccase-like protein YfiH of *A. media* strain WS (B224_5050) was a PPO.

### Catalase Is Responsible for PPO2 Activity in *A. media* Strain WS

Although we concluded that the identity of the gene responsible for PPO1 activity had been established, we were unable to identify the gene responsible for PPO2 activity by bioinformatic analysis. Thus, we employed LC-MS/MS to determine the identity of PPO2.Crude extracts of *A. media* strain WS were precipitated by addition of ammonium sulfate gradually until it was 80% saturated (Ito, 2000). Sediments at 45% of ammonium sulfate displayed the highest PPO2 activity, so we selected these for dialyzation and native-PAGE staining with 5 mM L-DOPA. The band exhibiting PPO2 activity was cut and characterized by LC-MS/MS.

Peptides from LC-MS/MS were searched against the complete genomic sequence of *A. media* strain WS and 55 proteins were identified in the band with PPO2 activity (Supplementary Table S2). All of these had been annotated in the genome database. From a literature search, we found that one protein (protein SeqID: B224_4951) annotated as “catalase” (CatA) may be involved in melanin formation. Catalase affects melanin formation in eukaryotes (Skamnioti et al., 2007; Maresca et al., 2008). It has also been demonstrated that hydrogen peroxide (H_2_O_2_) is generated in a series of chemical reactions coupled to the enzymatic formation of o-quinones by tyrosinase acting on monophenols and o-diphenols, and during the auto-oxidation of the o-diphenols and other intermediates in this pathway (Munoz-Munoz et al., 2009). Notably, catalase, superoxide dismutase, or peroxidase help remove the H_2_O_2_ generated by reaction R00009 (conversion of hydrogen peroxide to oxygen and water) and R00602 (conversion of methanol and hydrogen peroxide to formaldehyde and water; Mithani et al., 2011). Moreover, we found that the enzymatic reaction catalyzed by PPO in DOPA-melanin formation is very similar to R02670 (the conversion of 3-hydroxyanthranilate to cinnavalininate), which corresponds to catalase in the context of the KEGG tryptophan metabolism map (MAP00380; shown in Supplementary Figure S3). This analysis led us to suggest that catalase is involved in melanin formation, via PPO activity.

To determine whether CatA is important for PPO activity, we deleted the CatA-encoding gene *katE* in the wild type *A. media* strain WS and the Δ*yfiH* mutant strain and tested whether deletion of *katE* affected the PPO2 activity. As shown in **Figure 3**, the band corresponding to PPO2 disappeared in both the Δ*katE* and Δ*yfiH*Δ*katE* mutant, indicating that CatA is critical for PPO2 activity. Meanwhile, the PPO2 activity of both the mutant strains could be restored by supplying the *katE* gene in trans on the plasmid pBBR1MCS-5. Taken together, these results confirmed that CatA in *A. media* strain WS is responsible for the PPO activity of PPO2.

### Deletion of CatA Influences Total Pigmentation of *A. media* Strain WS

From these results, we hypothesized that YfiH and CatA have PPO activity and are important in the synthesis of DOPA-melanin in *A. media* strain WS. We next investigated their effect on melanin production.

The first step was to assess the importance of YfiH and CatA in the residual pigmentation in WS Δ*hppD* supplemented with tyrosine. *yfiH* and *katE* were deleted individually or in combination in the Δ*hppD* mutant of *A. media* strain WS to yield the mutants WS Δ*yfiH*Δ*hppD*, WS Δ*katE*Δ*hppD*, and WS Δ*yfiH*Δ*katE*Δ*hppD.* The production of melanin was measured in these mutants. Supplementary Figure S4 shows that the residual pigmentation was still present in these mutants, leading us to conclude that YfiH and CatA are not correlated with the residual pigmentation of *A. media* strain WS lacking HppD.

**FIGURE 4:**
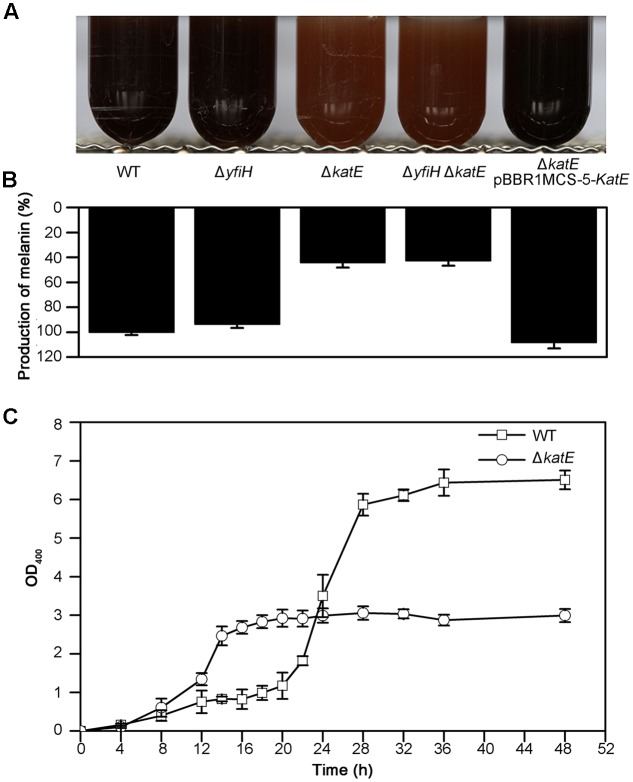
Characteristics of melanogenesis in *A. media* strain WS and the mutants WS Δ*yfiH*, WS Δ*katE*, WS Δ*yfiH*Δ*katE*, and WS Δ*katE* (pBBR1MCS-5-*katE*). **(A)** Photographs of cultures at 72 h in culture in LB media. **(B)** The melanin yield, as determined by OD_400_/OD_600_, after 72 h in culture. **(C)** Time course of melanogenesis in WS Δ*katE* (○) and *A. media* strain WS (wild-type; □). Note that melanogenesis started after 15 h in culture in the mutant compared to after 24 h in the wild-type.

We next examined the role of YfiH and CatA on the entire, intact pigmentation process in *A. media* strain WS. Deletion mutants of *yfiH*, *katE*, or of both genes were cultured in LB medium and the pigmentation process was compared to the wild-type strain. While deletion of *yfiH* did not affect pigmentation at all, deletion of *katE* caused about a 55% decline of pigmentation (**Figures 4A,B**). Melanogenesis began about 9 h earlier in the mutant than in the wild-type strain (**Figure 4C**).

Given that secondary metabolism (including pigment metabolism) may be repressed when cell viability is reduced, we thought it wise to assess cell viability in the CatA-deficient mutants. We measured the cell viability of both the wild-type strain and the CatA-deficient strain with the TTC method. The reduction of TTC to TPF occurs in living but not dead cells. As shown in Supplementary Figure S5, the curves of cell viability between the wild-type strain and WS Δ*katE* were similar, with viability declining in both after 20–22 h in culture. When the cells had been in culture from 14 to 20 h, the production of pigmentation in WS Δ*katE* increased minimally; although, the cells were clearly viable.

Expression of CatA from a plasmid pBBR1MCS-5-*katE* (containing the entire *katE* from *A. media* strain WS) in the Δ*katE* mutant restored pigmentation to the wild-type level (**Figures 4A,B**). From this series of experiments, we suggest that CatA is not important for the residual pigmentation in WS Δ*hppD.* However, it may regulate pigmentation in *A. media* strain WS via other mechanisms independently of its PPO activity.

### Catalase Activity of CatA Is Important for Pigmentation of *A. media* Strain WS

We next wished to elucidate the role of CatA in pigmentation. The first step was to confirm that CatA does, indeed, have catalase activity. Catalases have been reported in *Aeromonas* (Barnes et al., 1999; Rio et al., 2007). Sequence alignment showed that CatA from *A. media* strain WS had 98% amino acid sequence homology to catalases from other *Aeromonas* species. We assessed the catalase activity of CatA in the wild-type strain and the mutant strain deficient in CatA, using a catalase assay kit (Wrześniok et al., 2013). The results in **Table 2** show strong catalase activity (3.68 U/mL) in the cell lysates of the wild-type strain, but not in cell lysates of the Δ*katE* mutant. No catalase activity was detected in the cell supernatant from either strain (**Table 2**), indicating that CatA is an endoenzyme. These results confirmed that CatA has catalase activity.

**Table 2:** Catalase activity assay.

Samples	Protein concentration (μ g/mL)	Catalase activity
Cell supernatant of WS	nd	0.12 ± 0.07 U/mL
Cell supernatant of WS Δ*katE*	nd	0.05 ± 0.04 U/mL
Intracellular extracts of WS	nd	3.68 ± 0.28 U/mL
Intracellular extracts of WS Δ*katE*	nd	0.14 ± 0.09 U/mL
His_6_-tagged CatA	288.34 ± 7.88	1,570.02 ± 46.75 U/mg
His_6_-tagged Hpi	350.05 ± 3.24	463.82 ± 33.25 U/mg
His_6_-tagged Hpii	329.74 ± 5.52	173.89 ± 20.86 U/mg


*nd, not determined: protein concentration was not measured for the catalase activity assay of *A. media* strain WS (wild-type) and the mutant strain WS Δ*katE.**

**FIGURE 5:**
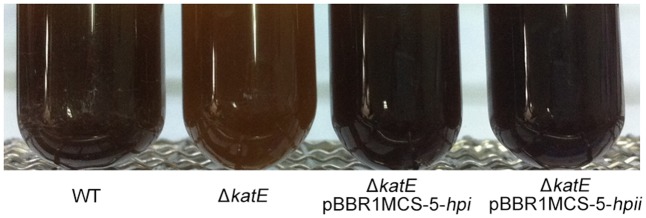
Hpi and Hpii from *E. coli* restore pigmentation to wild-type levels in WS Δ*katE* mutant strain. Photographs of wild-type *A. media* strain WS, WS Δ*katE*, WS Δ*katE* (pBBR1MCS-5-*hpi*), and WS Δ*katE* (pBBR1MCS-5-*hpii*) after 72 h in LB culture media.

In human melanocytes and cells of the rice blast fungus *Magnaporthe grisea*, both eukaryotic, the effect of catalase on hydrogen peroxide (H_2_O_2_) decomposition is well understood and correlates with melanin formation (Skamnioti et al., 2007; Maresca et al., 2008). Hydrogen peroxide generation has been observed in both the enzymatic reaction phase and the auto-oxidation phase of melanin biosynthesis (Munoz-Munoz et al., 2009). This information led us to speculate that production of hydrogen peroxide regulates pigment synthesis, and must be removed for maximum pigment production. To test this idea, we asked if expression of catalases from other bacteria in the WS Δ*katE* mutant could restore pigmentation to the wild-type level. Catalases Hpi and Hpii from *E. coli* BL21 (DE3), which show 47% amino acid sequence similarity with CatA from *A. media* strain WS, were chosen for this experiment (Supplementary Figure S6). As shown in **Figure 5**, expression of Hpi or Hpii from plasmids pBBR1MCS-5-*hpi*or pBBR1MCS-5-*hpii*in strain WS Δ*katE* restored pigmentation to the wild-type level. We interpreted these results to support our suggestion that oxidative stress regulates pigmentation of *A. media* strain WS.

### ROS Production during Pigmentation of *A. media* Strain WS

**FIGURE 6:**
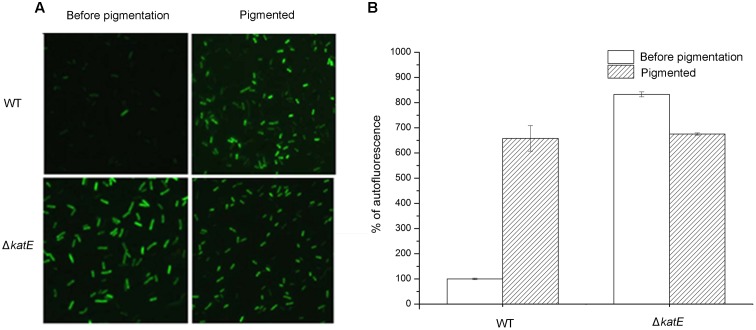
Qualitative and quantitative determination of oxidative stress caused by reactive oxygen species (ROS) during the period of melanin biosynthesis in *A. media* strain WS and WS Δ*katE*. **(A)** ROS assay, based on the fluorimetric probe dichloro-dihydrofluorescein diacetate (DCFH-DA), was determined by microscopy image analysis. **(B)** Quantification of ROS variation in the process of pigmentation in the two strains.

The results above led us to hypothesize that oxidative stress affects pigmentation in *A. media* strain WS, but the mechanism was unknown. To investigate the mechanism, we assessed the production of ROS during pigmentation in *A. media* strain WS, using the fluorimetric probe assay (DCFH-DA). Non-ionic DCFH-DA crosses cell membranes and is enzymatically hydrolyzed by intracellular esterases to non-fluorescent DCFH, which is then oxidized to fluorescent dichlorofluorescein (DCF) by hydrogen peroxide (LeBel et al., 1992). As showed in **Figure 6**, formation of fluorescent DCF was almost undetectable in the wild-type strain before pigmentation, suggesting that there was very little H_2_O_2_ produced. However, in the pigmented culture, a high level of DCF was detected in cells, indicating that H_2_O_2_ was generated during the pigmentation process. Unlike the wild-type strain, strong fluorescence of DCF was observed in the CatA deficient-mutant WS Δ*katE* before pigmentation, indicating that in the absence of CatA, ROS accumulated (**Figure 6A**). Interestingly, we found that the fluorescence signal of DCF in the WS Δ*katE* cells was higher before pigmentation than after pigmentation, suggesting that the ROS level of WS Δ*katE* before pigmentation (15 h after the start of the culture) was even higher than in the pigmented period (at 24 h). Oxidative stress-induced melanogenesis has been explored in some organisms such as in human B16F10 melanoma cells (Kim, 2012). The accumulation of ROS in WS Δ*katE* may explain the 9-h advance in production of melanin of the WS Δ*katE* mutant (**Figure 4C**). There are reports that melanin synthesis caused oxidative stress, for example, generation of H_2_O_2_ (Munoz-Munoz et al., 2009). Consistent with this, the fluorescence signal of DCF in the wild-type cells was similar to the WS Δ*katE* cells after pigmentation, which produced less than half of the melanin. These results suggest that accumulation of ROS induced production of melanin, but it may have negatively affected melanin synthesis at later stages.

### Dual-Activities Assay of the Active His_6_-Tagged CatA, Hpi, and Hpii *In Vitro*

**FIGURE 7:**
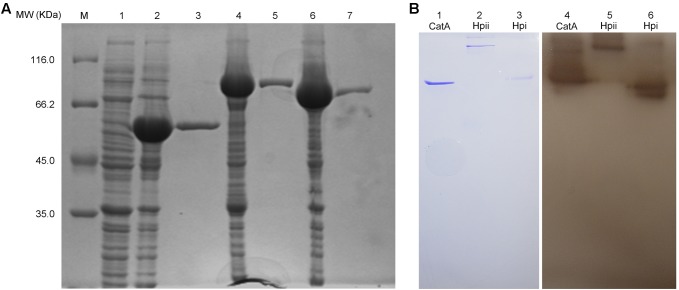
Purification and PPO activity assay of the active His_6_-tagged CatA, Hpi, and Hpii. **(A)** The proteins purified by Ni (II)-bound affinity chromatography were analyzed by SDS-PAGE (12%).Gel lanes: M, molecular weight marker; (1) the cell lysates from *E. coli* BL21(DE3) harboring pET-26b(+); (2) the cell lysates from *E. coli* BL21(DE3) harboring pET-CatA; (3) His_6_-tagged CatA purified; (4) the cell lysates from *E. coli* BL21(DE3) harboring pET-Hpii; (5) His_6_-tagged Hpii purified; (6) the cell lysates from *E. coli* BL21(DE3) harboring pET-Hpi; (7) His_6_-tagged Hpi purified. **(B)** PPO (PPO) activity analysis of purified proteins was carried out on native-PAGE. Gel lanes: (1) purified CatA stained by coomassie brilliant blue; (2) purified Hpii stained by Coomassie brilliant blue; (3) purified Hpi stained by Coomassie brilliant blue; (4) purified CatA stained by L-DOPA; (5) purified Hpii stained by L-DOPA; (6) purified Hpi stained by L-DOPA.

Earlier in this study, we demonstrated that CatA of *A. media* strain WS was important for PPO2 activity. We next wished to extend this finding, and confirm that the purified CatA protein has polyphenol activity as well as test catalases from other bacteria for PPO activity. To this end, CatA from *A. media* strain WS and catalases Hpi and Hpii from *E. coli*, were tagged with a His6 tag, purified, and analyzed by gel filtration. The purified proteins had a single peak with a molecular mass of 55, 80, and 84 kDa, respectively (**Figure 7A**). All His_6_-tagged proteins exhibited both catalase and PPO activity as we predicted (**Table 2** and **Figure 7B**). It appears that catalases are bi-functional enzymes and may constitute a new class of PPOs.

## Discussion

In this study, we confirmed the presence of DOPA-melanin in the pigment mixture produced by *A. media* strain WS, based on ESI-MS analysis. We demonstrated that the proteins YfiH and CatA possess PPO activity, and considered that they might catalyze the conversion of L-DOPA to DOPA-melanin. However, further analysis of *yfiH* and *katE* deletion mutant showed that YfiH and CatA were not essential for the residual pigmentation in WS Δ*hppD* supplied with tyrosine. Nonetheless, deletion of CatA caused pigmentation to occur earlier and reduced pigmentation that we believe was due to the accumulation of ROS in the wild-type strain. Expression of catalases from *E. coli* in the *katE* deletion mutant corrected the phenotype attributed to the deficiency of CatA, which we interpret as evidence that oxidative stress regulates pigmentation in *A. media* strain WS. We conclude that CatA has PPO activity in *A. media* strain WS and affects pigmentation through its scavenging activity.

*Aeromonas* is an opportunistic pathogen and may cause infections in invertebrates and vertebrates, such as fishes, birds, and human (Seshadri et al., 2006). Pigmentation is one of the key differential characteristics in the taxonomy for *Aeromonas* based on Bergey’s manual of systematic bacteriology. Categorization of *A. media* strain WS proteins using Web Gene Ontology Annotation Plot (WEGO) shows that approximately three hundred proteins are involved in the pigmentation process (Supplementary Figure S7), implying that pigmentation is a response to various environmental stimuli.

Although we demonstrated that pyomelanin is the major melanin constituent in *A. media* strain WS recently (Wang et al., 2015), many *Aeromonas* strains have also been proposed to produce DOPA-melanin, since the characteristic intermediate L-DOPA could be detected from the culture fluids of several melanogenic *Aeromonas* strains (Aurstad and Dahle, 1972; Donlon et al., 1983; Gibson and George, 1998; Wan et al., 2007). Here, we confirmed that DOPA-melanin was produced in *A. media* strain WS, although it was not a major contributor to pigmentation.

Production of DOPA-melanin is usually catalyzed by tyrosinase in bacteria, although in some cases it is catalyzed by laccase. Although we identified a tyrosinase, TyrA, from *A. media* strain WS (Wan et al., 2009), it does not seem to be important in melanin formation in this strain (Chai et al., 2012). Also, laccase has been reported in *A. hydrophila* WL-11 (Wu et al., 2010), but no homolog of this enzyme was present in the genomic sequences of *A. media* strain WS, *A. salmonicida* A449, *A. caviae*, or *A. veronii* B565 (Reith et al., 2008; Beatson et al., 2011; Li et al., 2011). In this study, we identified YfiH as a previously unrecognized laccase that displays PPO activity, but it also does not appear to be important for DOPA-melanin production or pigmentation in *A. media* strain WS.

In addition to YfiH, we found that the catalase CatA also exhibited PPO activity, which to our knowledge has not been previously reported in prokaryotes. Two catalases from *E. coli*, Hpi and Hpii, also displayed PPO activity. It may be, then, that catalases are bi-functional enzymes, similar to some hemocyanin and catechol oxidase/tyrosinase in eukaryotes (Yamazaki et al., 2004), two enzymes that belong to the PPO family and also exhibit catalase-like activity. Although catalase can convert L-DOPA to melanin, it is also does not seem to be important for DOPA-melanin production in *A. media* strain WS. These observations raise the question: What is responsible for DOPA-melanin production in *A. media* strain WS? One possibility is that there are additional enzymes in *A. media* strain WS that can convert L-DOPA to melanin. The fact that we only detected two bands with PPO activity make this suggestion unlikely, and also does not support the idea that these enzymes catalyzed the synthesis of DOPA-melanin by a new enzymatic reaction. Since L-DOPA has been reported to self-oxidize into melanin, the other possibility is that the residual pigmentation observed in the Δ*hppD* mutant cultured with tyrosine is due to the self-oxidation of L-DOPA.

Although CatA is not important for DOPA-melanin production, deletion of it reduced pigmentation of *A. media* strain WS. We suggest that this defect in pigmentation in the mutant was due to the accumulation of hydrogen peroxide, based on our finding that expression of catalases from *E. coli* in the Δ*katE* mutant restored melanogenesis. We propose that oxidative stress regulates melanogenesis in *A. media* strain WS.

Catalase is correlated with melanogenesis in some eukaryotic organisms, presumably due to its effects on hydrogen peroxide decomposition (Yamazaki et al., 2004; García-Molina et al., 2005). In human melanocytes, the physical shield of melanin and catalase activity, which is able to decompose H_2_O_2_, constitutes part of the organism’s protective strategies (Maresca et al., 2008). Catalase activity has been found inside melanosomes, presumably because of tyrosinase-related protein-1 (Halaban and Moellmann, 1990). The loss of function of the catalase CATB in the *catB* deletion mutant of *M. grisea* impaired melanization, believed to be due reduction of the laccase activity that is related to melanin biosynthesis (Skamnioti et al., 2007). These observations suggest that regulation of melanogenesis by catalase may be a widespread mechanism in both prokaryotes and eukaryotes.

Our previous work characterized the synthesis pathway of pyomelanin in *A. media* strain WS. The results of the current study are consistent with the idea that the level of ROS regulates the production of pyomelanin. We also showed that DOPA-melanin was produced in *A. media* strain WS, and we identified two PPOs. These findings should not only help us to understand the regulation of melanin production in *A. media* strain WS, but also enhance the pharmaceutical industry’s ability to incorporate melanin’s useful attributes into beneficial products. These attributes include anti-oxidant properties, protection from UV irradiation, and protection against viral infections. Melanin can also activate the immune system and maintain a proper balance of metal ions by binding to them. In addition, the intermediate of DOPA-melanin, L-DOPA, is a medicine for treatment of neurological diseases. Our findings and the numerous strains we have constructed should allow the engineering of *A. media* strain WS to produce melanin or L-DOPA in large quantities by industry.

## Author Contributions

BC and XC contributed to the conception and design of the study. BC, YQ, HW, XZ, JW, CW, and PZ performed the experiments and generated the data. BC and XC analyzed the data and wrote the paper.

## Conflict of Interest Statement

The authors declare that the research was conducted in the absence of any commercial or financial relationships that could be construed as a potential conflict of interest.

## Footnotes

1 http://www.matrixscience.com/
