# Supplementary material for: Identification of YfiH and the Catalase CatA As Polyphenol Oxidases of Aeromonas media and CatA as a Regulator of Pigmentation by Its Peroxyl Radical Scavenging Capacity
Source: Front Microbiol. 2017 Oct 5;8:1939. doi: 10.3389/fmicb.2017.01939 (PMC5633740; doi:10.3389/fmicb.2017.01939)
Supplement: Supplementary file 1 [file Data_Sheet_1.docx]

**Supplementary materials**


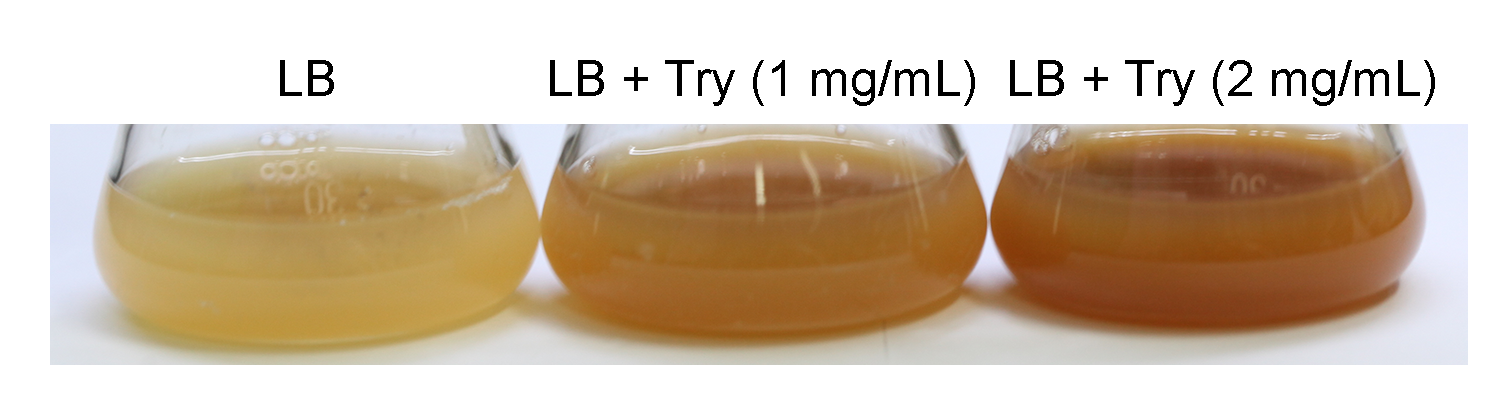


Figure S1. Photographs of cultures of the pyomelanin-deficient mutant WS Δ*hppD*, 72 h after culturing in media supplemented with different levels of tyrosine.


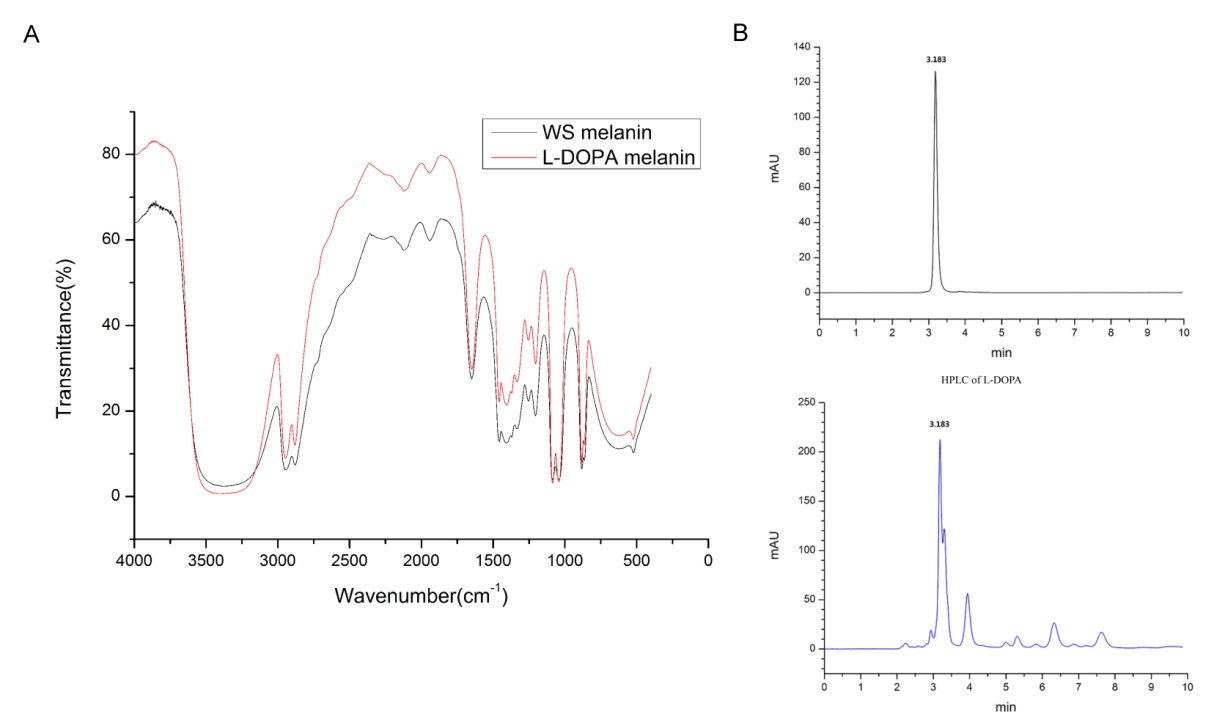


Figure S2. Fourier transform infrared spectroscopy (FT-IR) spectra of melanin and HPLC analysis of DOPA-melanin precursor from *A. media* strain WS. **(A)** The spectra of DOPA-melanin (red line) and *A. media* strain WS melanin (black line) were recorded over a range of 4,000–400 cm^-1^. **(B)** Detection of intermediates from cell-free culture filtrates of *A. media* strain WS, using synthetic L-DOPA as the standard.


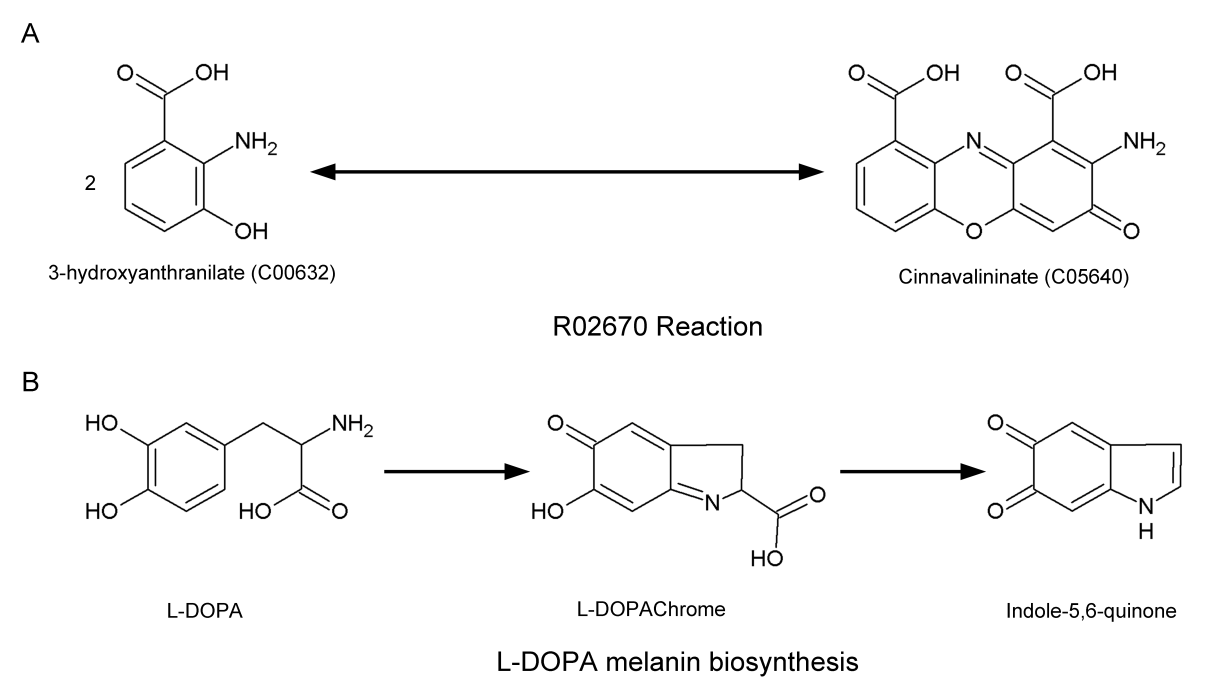


Figure S3. Comparison of reactions mediated by catalase and polyphenol oxidase in aromatic amino acid metabolism. **(A)** R02670 reaction catalyzed by catalase in tryptophan metabolism. **(B)** Reactions from L-DOPA to DOPA-melanin catalyzed by polyphenol oxidase in tyrosine metabolism.


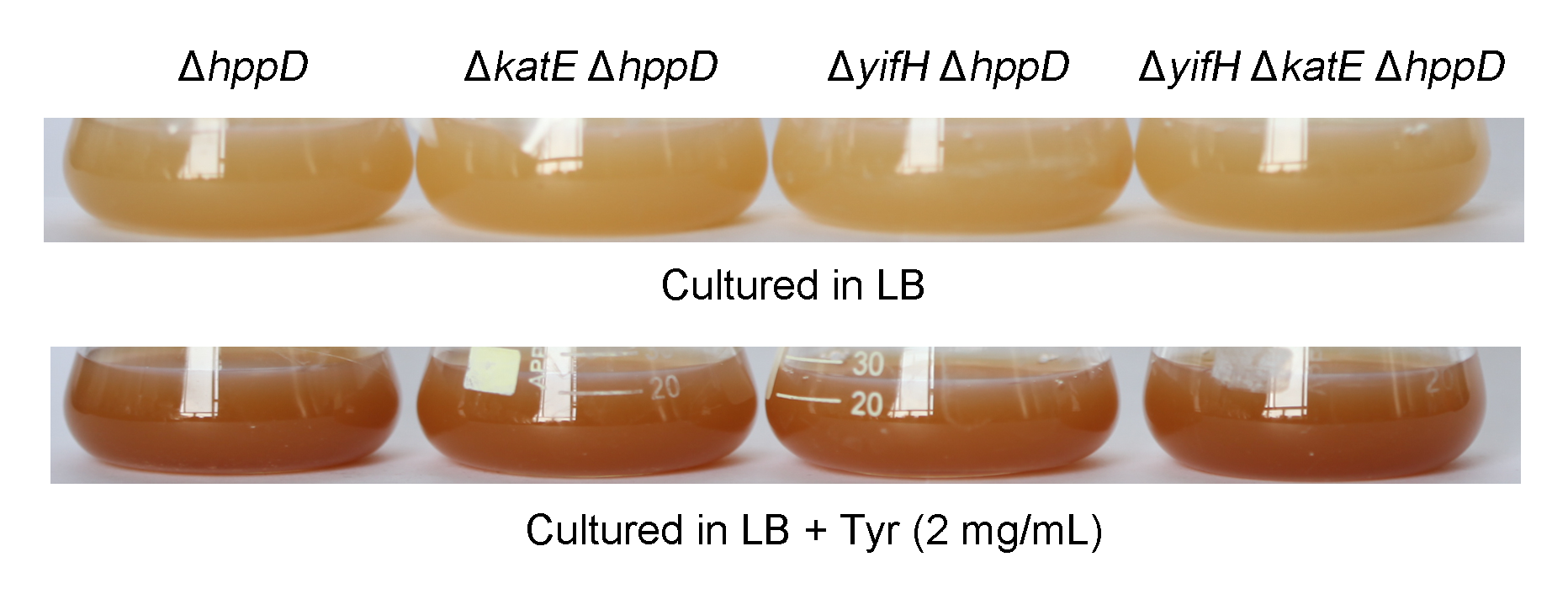


Figure S4. Photographs of HppD mutants of *A. media* strain WS after 72 h in culture, with or without supplementation with tyrosine. Note the influence of polyphenol oxidase activity on pigmentation.

**
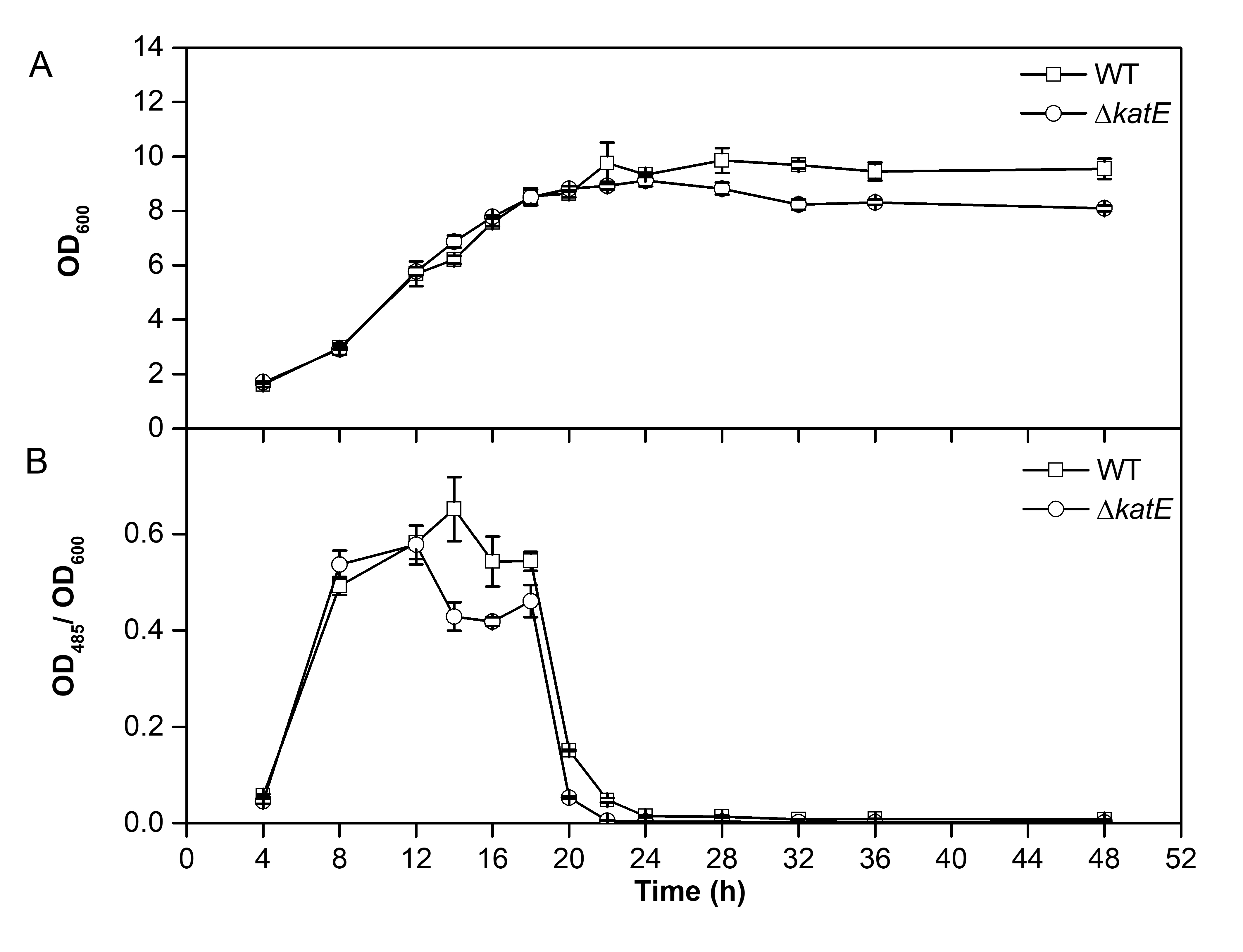
**

Figure S5. Growth and viability of *A. media* strain WS (□) and its mutant WS Δ*katE* (deficient in CatA; ○). **(A)** Growth as determined by OD at 600 nm. **(B)** Cell viability assay as assessed by the 2,3,5-triphenyltetrazolium chloride (TTC) method; cell viability was calculated using the OD485/OD600 value.


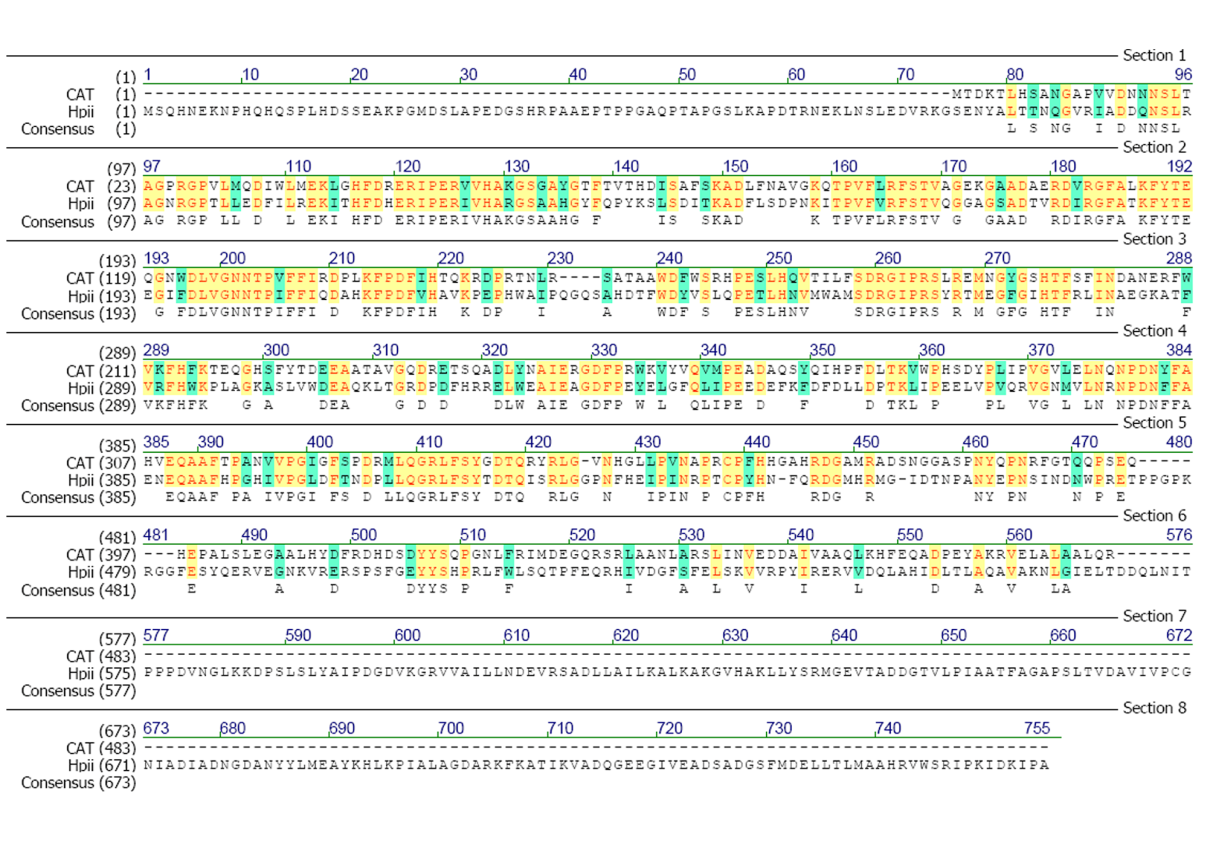


Figure S6. Alignment of CatA amino acid sequence from *A. media* strain WS with the corresponding KatE-domain protein sequence of Hpii from *E. coli* BL21 (DE3).


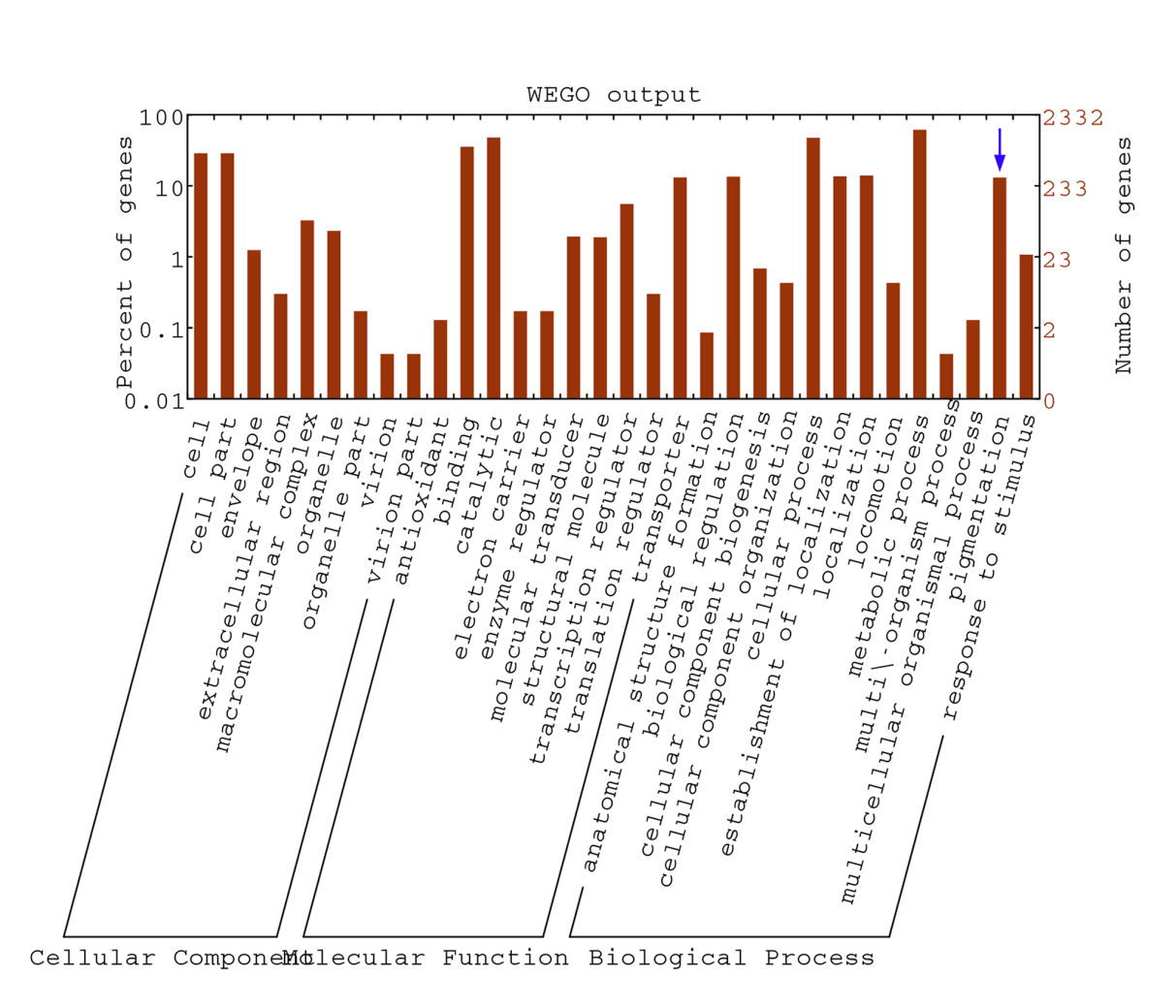


Figure S7. WEGO analysis of the complete genome sequence of *A. media* strain WS. Approximately three hundred proteins are predicted be involved in the pigmentation process (marked with blue arrow).

**Table S1 Oligonucleotide primers used in this study**

| Name | Sequence 5’→ 3’ | Usage |
| --- | --- | --- |
| **Primers used for construction integration plasmids** | | |
| yfiHUpF | GCCGTCGACAACCAGATAATCGAGGCCA | Forward primer for upstream of *yfiH* |
| yfiHUpR | ATTTCTAGACCTTTGAAGTGGGGGGCG | Reverse primer for upstream of *yfiH* |
| yfiHDnF | ATTTCTAGATGCAGGCCTGCCCGGCCT | Forward primer for downstream of *yfiH* |
| yfiHDnR | GCCGAGCTCTGGCCTGAGCGAAGGGGTC | Reverse primer for downstream of *yfiH* |
| katEUpF | ATTGTCGACGGTGCCCCCGTCGTCGAC | Forward primer for upstream of *katE* |
| katEUpR | GCCTCTAGAGAATTTCAGCGGGTCGCGA | Reverse primer for upstream of *katE* |
| katEDnF | ATTTCTAGA CTGCTGCCGGTCAACGCC | Forward primer for downstream of *katE* |
| katEDnR | GCCGAGCTCCGACCCGTTTGGCGTACTC | Reverse primer for downstream of *katE* |
| **Primers used for gene expression in pET-26b(+) vector** | | |
| CatAfor | CATATGACTGACAAGACCCTGCAC | Forward primer for *katE* amplification |
| CatArev | CTCGAGGCGCTGCAAGGCGGC | Reverse primer for *katE* amplification |
| Hpifor | CAT ATGAGCACGTCAGACGATAT | Forward primer for *hpii* amplification |
| Hpirev | AAGCTT CAGCAGGTCGAAACGGT | Reverse primer for *hpii* amplification |
| Hpiifor | CATATGTCGCAACATAACGAAAAG | Forward primer for *hpii* amplification |
| Hpiirev | CTCGAGGGCAGGAATTTTGTCAATCTTAG | Reverse primer for *hpii* amplification |
| **Primers used for identification of mutants** | | |
| yfiH-For | GGATCCCCGCCCTTGCAAG | Forward primer for *yfiH* mutant identification |
| yfiH-Rev | CCGTGAATGGAAGGAGTCAC | Reverse primer for *yfiH* mutant identification |
| katE-For | CCGCAGACGATCACTGTGA | Forward primer for *katE* mutant identification |
| katE-Rev | CGTAGAGATCGCGCAGGGG | Reverse primer for *katE* mutant identification |

**Table S2 The output of the MASCOT search results of polyphenol oxidase 2 (PPO2) activity protein**

| Score | Matches | Annotation on genome of WS |
| --- | --- | --- |
| 2623 | 82 (70) | tyrosinase |
| 979 | 43 (34) | riboflavin biosynthesis protein RibA |
| 859 | 31 (27) | catalase |
| 568 | 20 (17) | phosphoenolpyruvate synthase |
| 478 | 18 (17) | toluene tolerance protein Ttg2D |
| 476 | 16 (14) | basic membrane lipoprotein |
| 439 | 16 (15) | malate dehydrogenase |
| 384 | 8 (7) | phosphoglycerate kinase |
| 335 | 15 (12) | pflB protein |
| 326 | 18 (14) | glutamate synthase subunit alpha |
| 259 | 8 (7) | Enolase |
| 250 | 4 (4) | 2,3,4,5-tetrahydropyridine-2,6-dicarboxylate N-succinyltransferase |
| 235 | 5 (5) | alkyl hydroperoxide reductase |
| 210 | 7 (6) | D-ribose pyranase |
| 208 | 4 (4) | isocitrate dehydrogenase |
| 204 | 6 (6) | arcA-2 protein |
| 202 | 6 (5) | 50S ribosomal protein L1 |
| 179 | 8 (7) | aspartate ammonia-lyase |
| 151 | 9 (8) | Elongation factor G |
| 148 | 6 (6) | aconitate hydratase 2 |
| 146 | 4 (4) | DNA-binding protein HU-alpha |
| 120 | 6 (3) | 50S ribosomal protein L17 |
| 104 | 7 (3) | gap protein |
| 102 | 7 (2) | 4-aminobutyrate transaminase |
| 81 | 5 (4) | tkt protein |
| 62 | 1 (1) | malate dehydrogenase |
| 60 | 5 (4) | gltA protein |
| 59 | 6 (4) | succinyl-CoA synthetase subunit beta |
| 59 | 3 (2) | aceE protein |
| 54 | 3 (3) | oligopeptide ABC transporter, periplasmic oligopeptide-binding protein |
| 54 | 9 (8) | general secretion pathway protein A |
| 46 | 5 (3) | phosphoenolpyruvate carboxykinase |
| 46 | 1 (1) | rraA-2 protein |
| 46 | 5 (4) | phosphoribosylformylglycinamidine synthase synthetase subunit protein |
| 44 | 3 (2) | fumarate hydratase, class I |
| 34 | 9 (1) | Biosynthetic arginine decarboxylase |
| 32 | 1 (1) | acetylglutamate kinase |
| 32 | 1 (1) | F0F1 ATP synthase subunit alpha |
| 30 | 1 (1) | GMP synthase |
| 29 | 4 (1) | amino acid ABC transporter, periplasmic amino acid-binding protein |
| 28 | 1 (1) | D-3-phosphoglycerate dehydrogenase |
| 27 | 1 (1) | 30S ribosomal protein S7 |
| 24 | 1 (1) | potF protein |
| 23 | 1 (1) | rnr protein |
| 23 | 3 (1) | ketol-acid reductoisomerase |
| 20 | 1 (1) | carbamoyl phosphate synthase large subunit |
| 20 | 1 (1) | Uridine phosphorylase |
| 19 | 7 (1) | Fatty oxidation complex subunit alpha |
| 18 | 1 (1) | Alanine--tRNA ligase |
| 17 | 3 (1) | alkyl hydroperoxide reductase subunit F |
| 17 | 2 (1) | lysophospholipase L2 |
| 15 | 4 (1) | competence/damage-inducible protein CinA |
| 15 | 1 (1) | mce-related protein |
| 15 | 1 (1) | aceA protein |
| 14 | 19 (1) | membrane-fusion protein |
